# The transcriptome response of astronaut leukocytes to long missions aboard the International Space Station reveals immune modulation

**DOI:** 10.3389/fimmu.2023.1171103

**Published:** 2023-06-22

**Authors:** Daniel Stratis, Guy Trudel, Lynda Rocheleau, Martin Pelchat, Odette Laneuville

**Affiliations:** ^1^ Department of Biology, Faculty of Science, University of Ottawa, Ottawa, ON, Canada; ^2^ Bone and Joint Research Laboratory, Ottawa Hospital Research Institute, Ottawa, ON, Canada; ^3^ Department of Medicine, Division of Physiatry, Faculty of Medicine, University of Ottawa, Ottawa, ON, Canada; ^4^ Department of Biochemistry, Microbiology and Immunology, Faculty of Medicine, University of Ottawa, Ottawa, ON, Canada

**Keywords:** astronauts, spaceflight adaptation, leukocytes, immune gene expression, fluid shift, herpesvirus, transcriptome (RNA-seq)

## Abstract

**Introduction:**

Spaceflight leads to the deconditioning of multiple body systems including the immune system. We sought to characterize the molecular response involved by capturing changes in leukocyte transcriptomes from astronauts transitioning to and from long-duration spaceflight.

**Methods:**

Fourteen male and female astronauts with ~6-month- long missions aboard the International Space Station (ISS) had 10 blood samples collected throughout the three phases of the study: one pre-flight (PF), four in-flight (IF) while onboard the ISS, and five upon return to Earth (R). We measured gene expression through RNA sequencing of leukocytes and applied generalized linear modeling to assess differential expression across all 10 time points followed by the analysis of selected time points and functional enrichment of changing genes to identify shifts in biological processes.

**Results:**

Our temporal analysis identified 276 differentially expressed transcripts grouped into two clusters (C) showing opposite profiles of expression with transitions to and from spaceflight: (C1) decrease-then-increase and (C2) increase-then-decrease. Both clusters converged toward average expression between ~2 and ~6 months in space. Further analysis of spaceflight transitions identified the decrease-then-increase pattern with most changes: 112 downregulated genes between PF and early spaceflight and 135 upregulated genes between late IF and R. Interestingly, 100 genes were both downregulated when reaching space and upregulated when landing on Earth. Functional enrichment at the transition to space related to immune suppression increased cell housekeeping functions and reduced cell proliferation. In contrast, egress to Earth is related to immune reactivation.

**Conclusion:**

The leukocytes’ transcriptome changes describe rapid adaptations in response to entering space followed by opposite changes upon returning to Earth. These results shed light on immune modulation in space and highlight the major adaptive changes in cellular activity engaged to adapt to extreme environments.

## Introduction

The journey to space and sojourn in this extreme environment expose astronauts to health hazards such as cosmic radiations and microgravity (1). Short- and long-term spaceflight negatively affects most physiological functions: musculoskeletal, cardiovascular, respiratory, metabolic, endocrine, cognitive, gastrointestinal, microbial, genito-urinary, dermatological, ocular, and immune (1–3). A rapid physiological change occurring immediately upon entering space is the prompt redistribution of blood from the lower to upper part of the body (4). In response, plasma shifts toward extravascular tissues including the lymphatic system, resulting in diuresis and reduction in blood volume by ~10%-15% within the first days in microgravity (5). An opposite response occurs upon return to Earth; blood redistributes to the lower limbs, requiring an increase of total blood volume achieved by increasing fluid intake (6). These fluid shifts represent opposite physiological adaptations when transitioning to and from microgravity environments.

At the cellular level, plasma volume redistribution during spaceflight alters blood cell concentration –triggering mechanisms to restore homeostasis. An analysis of astronaut blood samples collected onboard the International Space Station (ISS) reported a ~17% elevation in white blood cell counts persisting during long-term spaceflight and accompanied by impairments of immune cell functions upon returning to Earth (7, 8). Early studies have also reported that, within 1 week in microgravity, red blood cell mass decreases ~10% (9). In a recent study, a temporal analysis including before, during, and after mission measurements of hemoglobin from 14 astronauts sojourning onboard of the ISS for ~6 months documented ongoing hemolysis in space (10). At the molecular level, studies have focused on the transcriptomic changes of astronauts during spaceflight. The National Aeronautics and Space Administration (NASA) Twin Study monitored simultaneously one twin experiencing spaceflight for ~1 year and compared data to his twin brother in the terrestrial environment. Transcriptional changes were found in multiple immune cell types including Peripheral Blood Mononuclear Cells (PBMCs), CD4 and CD8 T lymphocytes, and CD19 B lymphocytes. Gene expression in lymphocytes remained disrupted even after spaceflight (11). Those studies offered valuable insight into the molecular interactions occurring during spaceflight but suffer from small astronaut sample sizes and lack an integrated time-dependent analysis.

Genome-wide expression analysis provides a rich source of information to characterize molecular processes that underlie physiological adaptions associated with spaceflight (11). Peripheral blood cells are an ideal candidate to probe systemic effects due to their contact with multiple- organ systems through blood circulation. Transcriptional changes in circulating blood cells may thus reflect multisystem changes rich in information to assess astronaut health in response to the space environment and guide the design of personalized interventions. In the current study, we took advantage of the rare opportunity to sample a cohort of 14 astronauts onboard the ISS for ~6-month missions. We harvested the circulating leukocytes of ISS crewmembers before, during, and after spaceflight to establish the transcriptional composition and dynamic changes. This hypothesis-generating study combined an integrated time-course analysis followed by a time-point analysis at phase transitions to ISS missions. This approach captured the longitudinal changes in transcript levels and characterized the effect of transitioning to and from space, the adaptation in space, and state up to 1-year post-flight. We predict that the greatest transcriptional changes will occur at phase transitions entering spaceflight and returning to Earth, whereas minimal changes will be observed later in spaceflight.

## Methods

### Participant selection, study design, and ethics

Twenty astronauts scheduled to travel to the ISS voluntarily attended a presentation of the Marrow Adipose Reaction: Red Or White (MARROW) project approximately 1 year before an astronaut’s scheduled flight. Inclusion criteria included male or female astronauts that were non-smokers, without any metal implants, and were scheduled to remain on the ISS for a minimum of four months. Fourteen astronauts, 11 men (46.7 ± 7.3 years old) and three women (39.7 ± 2.1 years old) consented to participating in the study on the temporal analysis of the leukocyte transcriptomes from blood samples collected throughout the three phases of their mission: pre-flight (PF), in-flight (IF), and return to Earth (R). All participants gave informed consent and were monitored by a medical team at NASA. Ethics approval was obtained from the NASA Human Research Multilateral Review (#Pro1283), Johnson Space Center Institutional Review Board (JSC-IRB), European Space Agency Medical Board, Japanese Aerospace Exploration Agency, and the Ottawa Health Science Network Research Ethics Board #2009646-01H.

### Blood sample collection and storage

**Figure 1:**
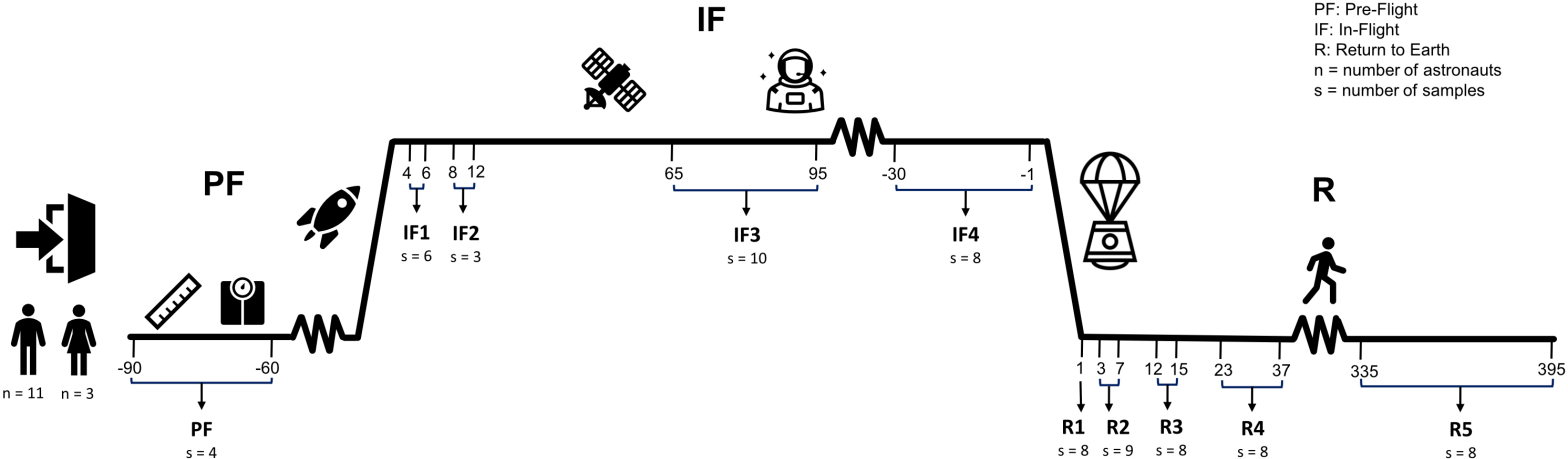
Experimental design. Fourteen astronauts (11 men and 3 women) sojourned aboard the ISS. Ten venous blood samples were collected from each astronaut throughout the three phases of their mission: one sample at pre-flight (PF) between 90 and 60 days before liftoff, four samples in-flight (IF) onboard the ISS (IF1: between days 4 and 6; IF2: between days 8 and 12; IF3: between days 65 and 95; IF4: 30 to 1 days before returning on Earth), and five samples upon return to Earth (R) (R1: day 1; R2: between days 3 and 7; R3: between days 12 and 15; R4: between days 23 and 37; R5: between days 335 and 395). “n” is the sample size of male and female astronauts that participated in the study. “s” is the number of RNA samples with libraries that passed quality metrics and used for sequencing at each timepoint.

Ten blood samples were collected from each astronaut: one sample at PF between 90 and 60 days before liftoff, four samples IF onboard the ISS (IF1: between days 4 and 6; IF2: between days 8 and 12; IF3: between days 65 and 95; IF4: 30 to 1 day before returning on Earth), and five samples upon return to Earth (R) (R1: day 1; R2: between days 3 and 7; R3: between days 12 and 15; R4: between days 23 and 37; R5: between days 335 and 395) (**Figure 1**). Approximately 4 ml of venous blood was collected after an overnight fast in a Vacutainer with plasma separator tube gel and 83 units of lithium heparin (#8362534, Becton Dickinson, Franklin Lakes, NJ, USA). Immediately after harvesting, blood tubes were inverted, centrifuged at 1,500g for 15 min at room temperature, and stored at −80°C. PF and post-flight blood samples were collected and centrifuged at the NASA Johnson Space Center in Houston Texas (USA) by a certified phlebotomist, and IF samples were collected, centrifuged, and frozen by crewmembers. The protocol for blood collection, centrifugation, and storage was the same for all blood samples including those collected onboard of the ISS. Frozen samples were shipped to our institution within 2–3 months after collection. A total of 139 blood samples were collected from 14 crewmembers during 12 different ISS missions (**Supplementary Figure 1**).

### Leukocyte capture, RNA isolation, library preparation, and sequencing

Frozen blood samples were thawed at room temperature for ~15 min, and leukocytes layered on top of the gel were resuspended by gentle inversion of the tube. Total RNA was isolated from leukocytes using the LeukoLOCK™ total RNA isolation system and manufacturer’s protocol (#AM1923, ThermoFisher Scientific, Waltham, MA, USA). Total RNA was suspended in 20 μ l of RNAse-free water, and quality was assessed using the Agilent BioAnalyzer 2100 (Model G2939B, Agilent, Santa Clara, CA, USA). All 139 samples had RNA integrity number (RIN) ≥8.0. RNA sequencing libraries were depleted of rRNA using the NEBNext^®^ ribosomal RNA (rRNA) Depletion Kit (Human/Mouse/Rat) (#6310L, Ipswich, MA, USA) or using the NEBNext® Ultra™ II Directional RNA (rRNA) Library Prep Kit for Illumina® (#E7760L, Ipswich, MA, USA). Library quality was assessed using the Agilent BioAnalyzer 2100, and 72 samples passed the quality metrics for sequencing including a concentration above 10 nM (**Supplementary Figure 1**). Details of the inventory of libraries included with randomly assigned astronaut identifiers and time points that passed quality metrics are provided in **Table 1**. Libraries (125 pM) were multiplexed and sequenced with 100-base paired-end reads to a depth of ~50 million reads per sample using the Illumina NovaSeq 6000 System (Illumina, San Diego, CA, USA). Library preparation, quality assessment, high-throughput sequencing, and de-multiplexing step were performed at Genome Quebec Innovation Center (Montreal, Canada).

**Table 1:** Sample inventory: libraries that passed quality metrics and used for sequencing.

Astronaut	Time-point									
n = 14	PF (n = 3)	IF1 (n = 6)	IF2 (n = 3)	IF3 (n = 10)	IF4 (n = 8)	R1 (n = 8)	R2 (n = 9)	R3 (n = 8)	R4 (n = 8)	R5 (n = 8)
a2073		*		*						
a2017				*						
a2029	*	*			*	*	*	*	*	*
a2005		*	*	*		*	*	*	*	*
a2096	*	*		*			*			
a2049			*	*	*	*	*		*	*
a2052				*	*		*	*	*	
a2020					*			*		*
a2068	*^1^		*	*	*	*	*	*		*
a2036		*			*	*	*	*	*	*
a2084				*		*	*		*	
a2091		*		*	*	*	*	*	*	*
a2057				*	*	*		*	*	*
a2031	*									

^1^Sample excluded as an outlier (**Supplementary Figure 1**).

### RNA-seq mapping

Reads were aligned to the publicly available human transcriptome and genome (GRCh38.84) using HISAT with default parameters (v2.0.13) (12). Transcript abundance calculations were performed using HTSeq with default parameters (v0.6.1) (13). For consistency with rRNA depletion library preparation, rRNA genes were excluded leaving an expression dataset that corresponded to 59,901 transcripts from coding and non-coding genes measured at the individual study time points for each astronaut.

### Sample quality control

Gene read counts for the 59,901 genes from each sample were normalized for sample read depth using *DESeq2’s* median of ratios (14), and the variance stabilizing log2 transformation (VST) was applied reducing variance of low read counts for principal component analysis. Principal component scores were calculated using the *prcomp()* function in R for all 72 samples. Samples were then plotted along PC1 and PC2 to visualize the variance explained by the individual astronauts and study time points. This showed significant deviation of the PF sample a2068.1 from other samples (**Supplementary Figure 2**). Therefore, this sample was treated as a technical outlier and excluded from further analyses leaving 71 samples for analysis *in silico*. All sequence analyses and result visualizations were conducted using the R environment (https://cran.r-project.org/doc/manuals/r-release/R-intro.html) and custom scripts.

### Differential expression analysis

Two separate differential expression analyses were completed with both using the same fixed-effect generalized linear model (GLM) within the DESeq2 package in R environment (14).


Model: read counts ~ replicate + sex + cumulative time in space + time + ε

This model measures the effect across time treating the “time” variable as categorical and included all 10 time points of the study. Confounding variables —sex and astronaut cumulative lifetime in space— were controlled while also accounting for repeated measures of biological replicates. The first differential expression analysis tested the temporal effect through applying likelihood ratio testing (LRT) on the “time” variable using the normalized read counts of all 59,901 genes. The temporal analysis was conceptually similar to analysis of variance (ANOVA) but instead represented an analysis of deviance (ANODEV) because DESeq2 estimates dispersion not variance. Adjustment for multiple comparisons was done through independent hypothesis weighting (IHW) using the *IHW* package in R environment (15). This method increased power by assigning weights to each hypothesis test while controlling for the false discovery rate using the Benjamini–Hochberg correction (16). Genes with adjusted p-values <0.1 were considered statistically significant and identified as gene candidates that were differentially expressed between any time point of the study.

For more specific insight into the IF and post-flight specific effects, differential expression analyses were done on a subset of time points. This used the same GLM as in the temporal differential expression analysis but instead applied the Wald’s test on log2 fold changes (LFCs) for significance testing followed by LFC shrinkage using the *ashr* method (17). Comparisons of selective time points were conceptually similar to a *post-hoc* analysis testing the effect of specific time points within the “time” variable. Adjustment for multiple comparisons was done using only the Benjamini–Hochberg correction (16). Genes with adjusted p-values <0.1 and LFC values >|0.5| were identified as gene candidates differentially expressed between a given time-point comparison.

### Transcriptome expression visualizations

**Figure 2:**
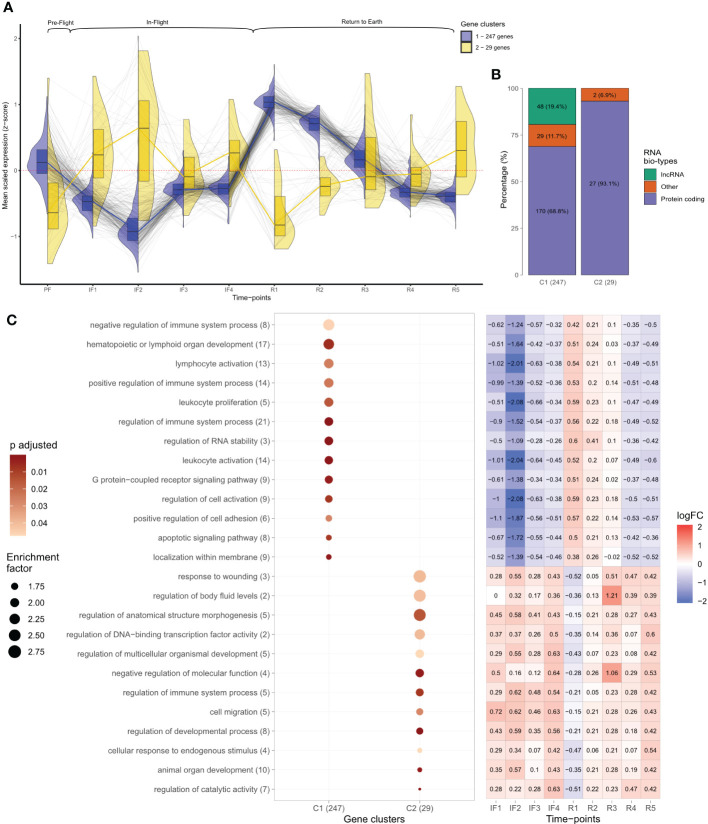
Temporal analysis of leukocyte transcriptomes before, during, and after spaceflight. **(A)** Gene expression levels plotted as scaled Z-scores across individual time points. Z-scores represent the average normalized read counts scaled across the 276 temporal differentially expressed genes at each individual time-point for the 14 astronauts. Black lines follow individual transcripts over time. Colored lines, split violin, and box plots represent the profiles of temporal gene clusters identified through hierarchical clustering. The number of genes identified in each cluster is indicated. Above brackets indicate the mission phases of time points. **(B)** RNA bio-type proportions for the two temporal gene cluster profiles displayed as stacked bar plots. RNA bio-type, gene counts, and percentages are indicated. The “other” category includes miscellaneous RNA, pseudogenes,and RNA to be experimentally confirmed (TEC). **(C)** Dot plot displaying the gene ontology (GO) terms obtained from the overrepresentation analysis (ORA) of temporal gene clusters across all mission phases. Terms with >1.5 enrichment in each temporal cluster were plotted with the number of genes mapping to that specific GO term indicated in brackets. The size of each dot is proportional to the enrichment factor (size scale) and the color represents the FDR adjusted p-values, where darker points have lower values (color scale). Enrichment corresponds to the ratio of mapped gene counts to a given GO term between each temporal gene cluster and the reference list of 15,410 genes. Enriched GO terms were grouped under “Biological Processes” at level 4. Using the Benjamini–Hochberg correction for multiple comparisons, p-values with false discovery rate (FDR) <0.05 were considered statistically significant. The heatmap displays the median log fold change (LFC) values throughout in-flight and post-flight time points relative to baseline for the genes associated with each GO term. The color bar represents values of log2 fold changes ranging from +2 (red) to −2 (blue).

The normalized read counts of gene candidates identified as differentially expressed between any of the 10 time points (temporal analysis) were averaged among all astronauts at each time point and scaled across genes as z-scores. These genes were then further analyzed to extract gene clusters displaying similar patterns of expression throughout the entire study. Briefly, the Euclidean distance between each gene candidate was calculated using their z-score scaled normalized read counts from each sample across time. These values were then hierarchically clustered using the *hclust*() function in R environment creating a tree dendrogram to visualize the gene clusters, which were resolved by a static tree cut (**Supplementary Figure 3**). Gene z-scores of temporal gene clusters were then plotted across time using lines and split violin plots overlayed by box plots to visualize the relative expression of genes over the course of the study (**Figure 2**).

As part of the temporal analysis, independent filtering of genes using *DESeq2* excluded genes with mean normalized read counts <45 resulting in a profile of 15,410 genes representing the expressed transcriptome across time. The normalized read counts for this profile of expressed genes were scaled separately as z-scores. Gene z-scores for the 15,410 genes were then plotted across time as violin plots (**Supplementary Figure 4**).

### RNA bio-typing

**Figure 3:**
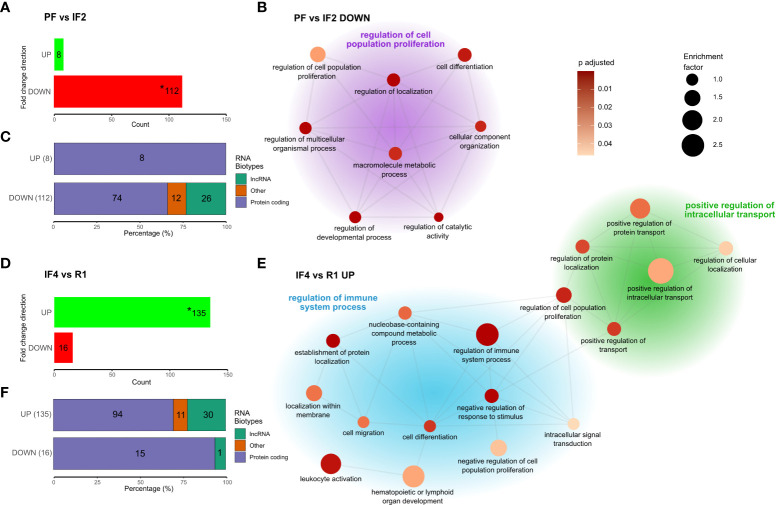
Genes differentially expressed at spaceflight phase transitions and functional enrichment. Cluster network of gene ontology (GO) terms was obtained from the phase-specific overrepresentation analysis (ORA) of 112 downregulated genes between PF and IF2 **(A, B)**, and 135 upregulated genes between IF4 and R1 **(D, E)** with stacked bar plots **(C, F)** displaying the RNA Bio-type proportions for these genes. RNA Bio-type, gene counts, and percentages are indicated. The “other” category includes miscellaneous RNA, pseudogenes, and RNA’s to be experimentally confirmed(TEC). Networks **(B, E)** illustrate semantic similarity of GO terms using REVIGO. The size of each dot is proportional to the enrichment factor (size scale) and color represents the FDR adjusted p-values, where darker points have lower values (color scale). Cluster headers are the GO term with the highest enrichment factor within that cluster. Enrichment corresponds to the ratio of mapped gene counts to a given GO term between each differentially expressed gene profile [PF vs. IF2 **(A)** and IF4 vs. R1 **(D)**] and the reference list of 15,410 genes. Enriched GO terms were grouped under “Biological Processes” at level 4. Using the Benjamini–Hochberg correction for multiple comparisons, p-values with false discovery rate (FDR) <0.05 were considered statistically significant. Asterisk (*) in bar plots **(A, D)** indicate the genes use in the enrichment analysis.

Gene candidate profiles identified from both temporal and time-point differential expression analyses were bio-typed according to the functionality of RNA transcribed from these genes. Bio-type annotation was done using the *biomaRt* package in R environment (18, 19), which utilized the Vega archive gene classifications (https://vega.archive.ensembl.org/info/about/gene_and_transcript_types.html). The relative proportions of RNA bio-types within each differentially expressed gene profile were then displayed as stacked bar plots (**Figures 2**, **3C, F**).

### Log fold change heatmap

**Figure 4:**
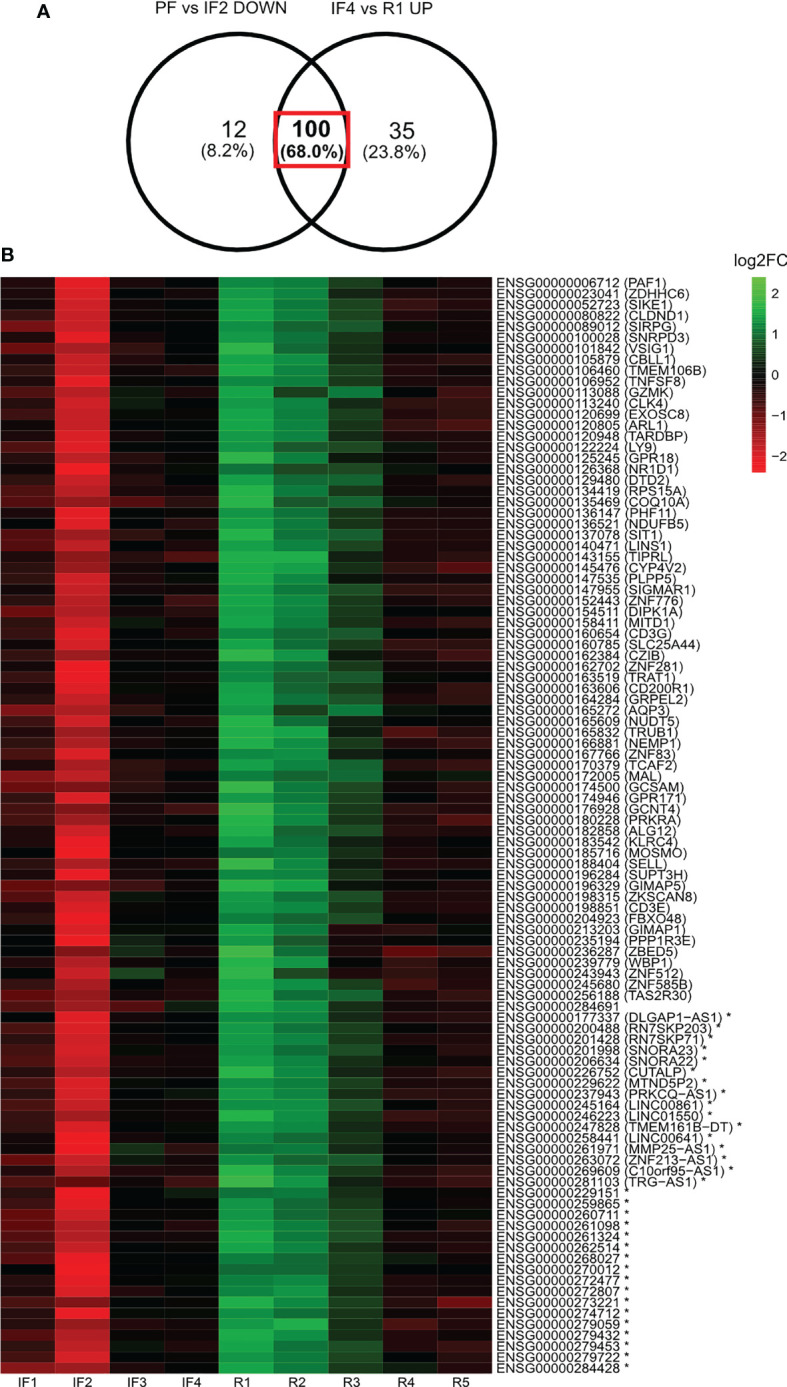
Log2 fold change of 100 genes displaying downregulation when reaching space and upregulation when landing on Earth after 6 months in space. Heatmap illustration of the log2 fold changes (Log2FC) of expression relative to pre-flight across all in-flight and post-flight time points for the 100 genes both downregulated when reaching space and upregulated when landing on Earth. The Venn diagram **(A)** indicates the profile of genes being displayed in the heatmap **(B)**. Expression levels of individual genes relative to pre-flight values are expressed across in-flight and post-flight time points as log2 fold changes displayed as a heatmap **(B)**. The color bar represents values of log2 fold changes ranging from −2 (red) to +2 (green). Gene identities are shown by their *ensembl* ID and corresponding *HGNC* symbol (if applicable) in brackets. Asterisk (*) indicates non-coding genes.

The LFC values relative to PF were calculated for a subset of gene candidates identified from the differential expression analysis of selective time points. This subset of genes was identified from the Venn diagram overlap between the differentially expressed gene profiles for PF vs. IF2 and IF4 vs. R1 (**Figure 4**). LFC values relative to PF were then displayed as a heatmap across all IF and post-flight time points along with their gene identities (**Figure 4**).

### Enrichment analysis

For insight into the broader biological functions of the differentially expressed gene profiles, functional enrichment of leukocyte transcriptomes using two separate overrepresentation analyses (ORAs) (20) of gene ontology (GO) terms was performed: one is to assess the temporal effect across all time points using the differentially expressed gene clusters and the other is to assess the spaceflight phase transitions using the differentially expressed genes from the selective time-point comparisons. A custom R script was used to detect significantly overrepresented GO terms between the list of differentially expressed gene profiles and the 15,410 expressed genes used as the reference list. ORA utilized clusterProfiler 4.0’s *groupGO()* function (21) to map genes to their associated level 4 GO terms grouped under “Biological Processes”. A Fisher exact test was applied to test for significantly overrepresented GO terms between genes and the reference list. After adjustment for multiple comparisons using the Benjamini–Hochberg correction, GO terms with FDR p-values <0.05 were considered statistically significant.

Significant GO terms from the temporal gene clusters with >1.5 enrichment were displayed onto a dot plot. The median LFC values relative to PF for the genes mapping to a specific GO term were displayed as a heatmap across all IF and post-flight time points (**Figure 2**). For differentially expressed genes between specific time points, significant GO terms were summarized by cluster networks based on semantic similarity (**Figures 3B**) using REVIGO via a web browser (https://revigo.irb.hr) (22). The GO term cluster networks were further processed for clarity and aesthetics using Cytoscape (v3.9.1.0) (23).

## Results

### Temporal analysis of leukocyte transcriptomes

To report the effects of space mission to the ISS, we determined the composition of astronauts’ transcriptomes and measured differential expression over time using a GLM within the *DESeq2* package. LRT of all 10 time points identified 276 genes differentially expressed between any time point of the study. In addition, we found 15,410 genes represented the expressed transcriptome of astronauts over all 10 time points and corresponded to genes with average normalized read counts >45 according to *DESeq2’s* independent filtering threshold.

#### Transcriptome changes at transition to and from space

Temporal changes for the 276 differentially expressed genes are shown in **Figure 2**, which plots the mean transcript level for each gene across all 10 time points of the study. Transcript levels were reported as z-scores, which centered and scaled the normalized read counts for each gene by the mean and standard deviation across the 276 genes, ensuring comparable scaling and visualization for all genes. A positive z-score was interpreted as above average gene expression, a negative z-score indicated below average gene expression, and zero corresponded to average levels. Hierarchical clustering identified two gene clusters (C) based on the similarity in z-score changes over time (**Figure 2**; **Supplementary Figure 3**). The two gene clusters consisted of 247 (C1) and 29 (C2) genes, respectively, and mirrored each other; average levels changed in opposite directions at individual time points. Both showed inverse patterns of expression changes at transition to space and at the transition back to Earth (**Figure 2**). Specifically, gene C1 was characterized by decreased expression after 8–12 days in space (IF2) followed by increased expression on day 1 after return to Earth (R1) (**Figure 2**). Conversely, gene C2 displayed increased expression transitioning to space and decreased expression at return to Earth (**Figure 2**). Gene expression changes in C1 were less variable than C2 as shown by the narrower spread in violin plots and smaller interquartile ranges (IQR) of box plots (**Figure 2**).

#### Protein-coding genes dominate temporal gene clusters

Bio-typing of gene RNA revealed distinct bio-type proportions of the two temporal gene clusters. C1 consisted mostly of protein-coding genes (68.8%), 19.4% long non-coding RNAs (lncRNA), and 11.7% genes coding for other various RNA biotypes (**Figure 2**). C2 genes consisted mostly of protein-coding genes (93.1%), zero lncRNA, and 6.9% other RNAs (**Figure 2**).

#### The two temporal gene clusters differ in biological function

Functional enrichment of the two differentially expressed gene clusters across all 10 time points produced terms describing different biological functions. Shown in a dot plot are the GO terms from each temporal gene cluster with an enrichment >1.5 (**Figure 2**). In C1, seven of the top eight most enriched terms described immune system and leukocyte functions. Among these, the terms “regulation of immune system”, “lymphoid organ development”, and “leukocyte and lymphocyte activation” had the largest gene counts (**Figure 2**). In contrast, C2 was composed of terms describing diverse biological processes including anatomical structure and development and molecular regulation such as “DNA-binding transcription factor” (**Figure 2**). Interestingly, the second most enriched term of C2 was “regulation of body fluid levels” (**Figure 2**). The heatmap in **Figure 2** displays the median LFC values relative to baseline for the genes associated with a given GO term throughout IF and post-flight time points. LFC values reiterated the opposite profiles of changes characterizing the two clusters of genes: inverse pattern of expression at IF and post-flight transitions (**Figure 2A**).

#### Gene expression converges toward average levels after long-duration exposure to space

Gene expression between 2 and 6 months IF (IF3 and IF4) converged toward average levels. This pattern was observed for the 276 temporally differentially expressed genes (**Figure 2**) and was also evident with the profile of 15,410 genes obtained from independent filtering in DESeq2 (**Supplementary Figure 4**). The spread of average transcript levels for all expressed genes at IF3 and IF4 displayed the smallest IQR compared to all other time points (**Supplementary Figure 4**; **Supplementary Table 1**).

### Leukocyte transcriptome at spaceflight phase transitions

To assess the effects of space transitions on astronauts’ transcriptomes, four time-point comparisons were selected on the basis of the most important changes in transcript levels displayed in the temporal profiles of clusters (**Figure 2**). The time-point comparisons included space phase transitions (PF vs. IF2 and IF4 vs. R1), transcriptional convergence after long-duration in space (IF3 vs. IF4), and 1 year after return from space (B vs. IF5). The differential expression results for PF vs. IF2 and IF4 vs. R1 are shown in (**Figures 3A, B**, respectively). All four differential expression results are summarized in **Table 2**.

**Table 2:** Summary of selective time-point differential expression results.

Test^1^	Differentially expressed genes (DEGs)		
	Total	Upregulated^2^	Downregulated^3^
PF vs. IF2	120	8	112
PF vs. R5	0	0	0
IF3 vs. IF4	0	0	0
IF4 vs. R1	151	135	16

^1^Pairwise time-point significance testing using the Wald’s test and ashr log_2_fold change (LFC) shrinkage.

^2^LFC > 0.5

^3^LFC < −0.5

#### Differential expression was strongest during transitions to space and return to Earth

Comparing transcriptomes at PF and IF2 time points, we identified 112 downregulated genes and eight upregulated genes (**Figure 3**). The return to Earth was associated with 16 downregulated genes and 135 upregulated genes differentially expressed between IF4 and R1 transcriptomes. Substantial gene expression changes at space transitions were dominated by genes downregulated during early spaceflight (IF2) and upregulated during the return to Earth (R1). These results confirm the decrease-then-increase pattern of gene C1 identified in the temporal gene cluster analysis (**Figure 2**). In addition, RNA bio-typing of the differentially expressed genes at space transitions (PF vs. IF2 and IF4 vs. R1) replicated results from the temporal gene clusters with protein coding as most represented RNA (**Figures 3C**).

#### Space transition responses differ in biological function

Enrichment analysis of the 112 downregulated genes between PF and IF2 and 135 upregulated genes between IF4 and R1 identified biological functions differing between the transitions to and from space. GO terms are summarized in a cluster network on the basis of semantic similarity shown in **Figures 3B, E**. The transition to space enriched terms is related to cellular growth such as “cell population proliferation” (most enriched), “cell differentiation”, and “cellular component organization” (**Figure 3**). In contrast, the return to Earth resulted in two clusters of enriched terms both describing different biological processes than the transition to space (**Figure 3**). One cluster consisted of terms related to cellular transport such as “intracellular transport” and “protein transport and localization” (**Figure 3**). The other cluster included terms describing the regulation of immune system processes such as “leukocyte activation” and “lymphoid organ development” (**Figure 3**).

#### One hundred genes were both downregulated when reaching space and upregulated when landing on Earth

Among the 112 downregulated genes when reaching space and 135 upregulated genes when returning to Earth, 100 were the same genes (**Figure 4**). **Figure 4** lists the 100 genes along with a heatmap displaying the LFC values relative to PF for each gene. The three most represented gene families were Zinc-Finger Protein (ZNF) genes (n = 6), Cluster of Differentiation (CD) genes (n = 3), and Long Intergenic Non-Protein Coding (LINC) RNA (n = 3).

### Leukocyte transcriptome in-flight and 1-year post-flight

#### No differential expression occurs during late in-flight

Our results found zero genes differentially expressed between 65–95 days IF and 30–1 day prior to return to Earth (IF3 vs. IF4) (**Table 2**). The convergence toward no changes later in flight is also evident from standardizing scaled expression to z-scores. The distribution of mean scaled expression (z-scores) for all genes at each time point are shown in **Figure 2**; **Supplementary Figure 4**. Late IF time points corresponding to IF3 and IF4 had the lowest IQRs compared to all 10 time points (0.29 and 0.32, respectively) (**Supplementary Table 1**).

#### Transcriptome 1-year post-flight is similar to pre-flight

Analysis between PF and 1-year post-flight time points (PF vs. R5) revealed zero differentially expressed genes (**Table 2**). From the 15,410 expressed genes, transcriptional variability between PF and 1 year after returning from space appeared similar on the basis of the spread of violin plots and IQR (**Supplementary Table 1**; **Supplementary Figure 4**). However, **Figure 2** shows C1 and C2 having reversed expression 1-year post-flight when compared to PF. C1 genes had above average expression PF but had below average expression 1-year post-flight, and vice versa for C2.

## Discussion

We analyzed the leukocyte transcriptome of 14 female and male astronauts before their launch to space, upon reaching the ISS, in space for 6 months, at egress to Earth, and up to 1 year after landing. Differential expression was measured using an integrated time-course analysis followed by a focused analysis of mission phase transition time points. The salient findings were as follows (1): temporal analysis identified the decrease-then-increase expression pattern at transitions to and from space as the main profile of change with immune system processes most represented; (2) phase transition analysis identified downregulated genes mainly associated with “regulation of cell population” and upregulated genes at the return to Earth associated with “regulation of immune system process”; (3) 100 genes were both downregulated when reaching space and upregulated upon returning to Earth; and (4) transcript levels converged toward average levels displaying no differential expression between ~2 and ~6 months IF.

### Differential expression at mission phase transition

The first analysis of the leukocyte transcriptomes provided an overview of the relative transcriptional changes occurring at 10 time points across the three phases of a space mission: PF, IF, and R. Astronauts’ leukocyte transcriptomes showed opposite directions of gene expression changes upon reaching the space environment compared to the return on Earth. Cluster analysis grouped the differentially expressed genes into two clusters characterized by major changes in opposing directions: (C1) decrease-then-increase and (C2) increase-then-decrease.

The biological processes represented among the 247 genes from C1 were mainly specialized leukocyte functions and immune system processes. Temporal transcriptome data indicated a reduction of immune functions when transiting to space and the opposite when returning on Earth: an increase of immune function. Our findings are consistent with previous reports of decreased immunity in space including reductions in T- cell function, NK- cell function, altered plasma cytokine profiles, and persistent inflammation (7, 8, 24–26). Our analysis at both transition to a different gravitational environment revealed novel genes and pathways not previously documented in astronauts traveling to space. The decreased expression of the cell surface receptor *CD3E* and *CD3G* genes, both members of the CD3–T- cell receptor complex, is likely contributing to the reduced immunity while in space. The CD3 complex is involved in the recognition of antigens and subsequent signal transduction, leading to the activation of T lymphocytes (27, 28). The reduction of CD3 expression in response to microgravity was previously observed *in vitro* in a human cell line of T lymphocytes and Jurkat cells exposed to microgravity (29). Our data from astronauts’ leukocytes provide additional evidence for the CD3 complex dynamic response to microgravity and changes occurring within the first few days after transitioning to and from space. Considering the rapid changes of CD3 complex gene expression, adaptive immunity such as the response to foreign antigens is likely affected by changing microgravity environments, rather than innate immune systems (30). The impact of the differential expression on the adaptive immune system can not be excluded as both immune systems are highly interconnected and previously documented to be impacted by microgravity (31, 32). Few studies have examined the dynamic changes of markers of adaptive and innate immune response throughout ISS missions, and published data are from either short-duration missions (8–15 days) or to comparisons between pre- and post- flight (33, 34). A comprehensive study of eight astronauts sojourning ~6 months onboard of the ISS reported no or very little effects on B cell number, phenotype, and antibody output after returning on Earth (35). In this study, total B cells and immunoglobulin A increased after 90 days in flights and returned to baseline at return day. Our data on leukocytes’ transcriptome agree with the previous observations of transitional changes in immune cells while transitioning to and from space and the return toward baseline levels later after returning to Earth.

The lower number of genes in C2 (n = 29) limited the conclusions for enrichment analysis and identification of represented biological processes. Of interest, the biological term “regulation of body fluid” represented in the short list of genes in C2 displayed a pattern of upregulation when reaching space. The gene *SLC4A1* associated with the term “regulation of body fluid” encodes for an anion exchanger protein localized in the plasma membrane of erythrocytes and mediates carbon dioxide transport to the lungs (36). Increased expression of *SLC4A1* gene when reaching space may respond to the increase of carbon dioxide levels in conditions of low red blood cell mass, with the latter being previously documented in astronauts (10). The gene *AQP3* with changes in opposite directions at both phase transitions functions as a water and urea exit mechanism of antidiuresis in collecting duct cells —a mechanism regulating body fluids (37). Therefore, reaching space promoted leukocyte gene expression related to basic housekeeping cell functionality as well as specific space adaptations like headward body fluid shifts leading to loss in plasma volume and hemoconcentration (38). Restoring blood cells concentrations to homeostatic levels requires a decrease in the number of circulating leukocytes and red blood cells whose population is decreased by ~10% in the first 10 days in space (39). Therefore, in addition to immune functions, the leukocyte transcriptome identified cellular functions and physiological systems affected by spaceflight.

The opposite directions of expression changes in the gene clusters at space transitions replicated the results obtained from participants subjected to a microgravity analogue (40). The 6° head- down tilt bedrest model replicates the microgravity component of spaceflight with many of the physiological changes happening in space including fluid shift, muscle atrophy, bone loss, and hemolysis (41–44). Transcriptome composition changed in opposite directions at transitions between ambulation and bedrest and between bedrest and re-ambulation in 20 healthy participants submitted to 60 days of bedrest (40). While the space missions were longer with an average of 6 months compared to the 60 days period in bed, the transcriptome changes at phase transition coincided. Comparable changes in the leukocyte transcriptome may, therefore, indicate a characteristic response to the negative mechanotransduction, inactivity, and fluid shift brought about by prolonged exposure to both bedrest and space. Leukocyte transcriptomes are therefore highly sensitive to changes in the gravity vector and appear to mount an adaptive response toward restoring homeostasis.

The next characteristic of leukocyte transcriptome temporal changes observed was the transcriptional convergence toward average levels displaying no differential expression after 2 months of space exposure. This is a novel finding revealed through the temporal analysis. The biological meaning is unclear but indicative of global mechanisms yet to be identified that limit variations of mRNA levels in leukocytes in space. Interestingly, the gene expression convergence of astronauts replicated the results of participants to the 60-day bedrest study (40), supporting that gene expression convergence is related to inactivity and redirected gravity isolated from other space specific stressors. In addition, this might be compatible with a generalized loss of specialized cell functions upon removal of normally oriented gravity and activity. The lack of mechanotransduction and inactivity would then focus cellular activity on core housekeeping functions.

The comparison of transcriptomes between PF and 1-year post-flight showed that the two gene clusters were reversed in expression. This may suggest that some molecular space adaptations acquired while living in space for 6 months were maintained for at least 1 year after return to life on Earth. This may bear physiological significance given the ~20% increases in hemolysis in the same astronauts 1 year after returning from space (10, 39).

### Shift in biological functions at spaceflight transitions to and from microgravity

Transiting to and from microgravity was associated with the differential expression of 120 and 151 genes from the reference list of 15,410 genes expressed in leukocytes. The majority (93.3%) of the differentially expressed genes when reaching space were down regulated and 95.7% were up regulated when returning. Differential expression measured at mission transitions is consistent with the temporal profile of C1 characterized by down- and up- expression. Downregulated genes identified between PF and early IF were associated with the biological term “regulation of cellular population proliferation”. This transcriptomic response is consistent with the head ward fluid shift and subsequent hemoconcentration of blood cells occurring when entering space (38). A decrease in circulating red and white blood cells restores blood cell concentrations to maintain homeostasis, consistent with the downregulation of genes involved in cellular proliferation (10). A suppression of blood cell proliferation represents an adaptation to the reduced blood volume in space.

At transition from space to Earth, transcriptomes were characterized by an up regulation of expression, opposite to changes measured when reaching space. Enrichment analysis of the upregulated genes between late IF and return to Earth resulted in biological processes describing the regulation of immune system, leukocyte activation, and lymphoid organ development. Returning to Earth’s surface gravity after ~6 months in microgravity reversed the down regulation of genes involved in immune processes. Many immune alterations persist during long-duration spaceflight (8). Reactivation of immune- related genes in response to the re-entry to Earth is needed to reverse immune dysregulation occurring during spaceflight. The composition of the leukocytes’ transcriptome was influenced by the transition to the different gravity environments.

Of the 112 genes downregulated early IF, 100 (89.3%) of those same genes were upregulated immediately upon return to Earth. This means that the same genes responded to both transitions to and from microgravity despite the occurrence of different physiological changes. To our knowledge, this represents a novel finding. Most differentially expressed genes at space transitions coded for proteins with the second most important being long non-coding RNAs. The notable modulation of transcription factors (ZNF and CD) and lncRNAs that regulate expression of downstream target genes may explain why the same differentially expressed genes are regulating different physiological responses. Zinc Finger proteins are transcriptions factors that have a wide range of molecular functions including DNA recognition, RNA packaging, transcriptional activation, regulation of apoptosis, protein folding and assembly, and lipid binding (45). Changes in ZNF expression would potentially alter these molecular processes that would then manifest at the cellular and physiological levels. For instance, we identified Zinc-Finger Antiviral Proteins (ZAP) ZNF776, ZNF585B, and ZNF83 as downregulated during early spaceflight and upregulated upon return to Earth. ZAPs help prevent the spread of viruses by targeting viral mRNA (46). The downregulation of these genes when reaching space corresponds to reported reactivation of herpesvirus in astronauts during spaceflight (27). Whereas, the following upregulation of ZAPs when returning to Earth may be a response to suppress the replication of herpes viral particles.

## Contributions and limitations

Our access to unique astronauts’ blood samples and RNA analysis of the leukocytes’ transcriptome using high- throughput sequencing technique represents the strength of this study. The finding of genes responding to both the transition to and from space with decreased and then increased profile of changes that related to immune processes represents a novel finding. Our study also identified additional expression changes at phase transitions in genes unrelated to specific immune functions, such as cell population regulation. This provides evidence of changes at the molecular level by which the body adapts to the headward fluid shift observed in microgravity environments. This study bears a number of limitations. Blood draws were taken at 10 different time points throughout astronaut missions; changes of interest to establish the onset of transcriptional convergence in space timed in-between blood draws may have been missed. Technical limitations onboard the ISS hampered sample acquisition, processing, and analysis. For instance, blood samples were collected within a window of days rather than on a specific day that introduced variability. Leukocyte and RNA isolation were not possible on the ISS and blood samples were frozen at −80°C for their journey back to Earth, leading to cell lysis and RNA degradation. This resulted in samples with inadequate RNA quality for sequencing, which were rejected, leading to an unbalanced final sample size. In addition, a potential contribution of altered leukocyte subpopulations to gene differential expression can not be excluded. RNA sequencing removed ribosomal RNA and was biased toward protein-coding genes; changes in other RNA biotypes would have been missed. The limited sample size and heterogeneous cohort of 14 astronauts with unequal sex distribution limited statistical power and prevented sex-specific comparisons.

## Conclusion

The analysis of transcriptome composition identified changes during the transitions to and from space characterized mainly by a decrease and an increase of transcript levels respectively. When reaching space, the transcriptomic changes are indicative of decreased immune functions and increased basic cellular activities linked to adaptive changes. The transcriptomic changes egressing back to Earth were in opposite direction —increased expression, mainly for genes related to the immune system. These results shed light on immune modulation in space, the timing of differential expression at transition to and from space, and highlight the major adaptive changes in leukocyte activity engaged to adapt to extreme environments.

## Data availability statement

The datasets presented in this article are not readily available because of ethical and privacy restrictions. Requests to access the datasets should be directed to the corresponding author/s. Aggregated data to understand and assess the conclusions of this research are available in the figures and Supplementary Tables. Aggregated data (read count tables and metadata) have been deposited in NASA’s Life Sciences Data Archives (LSDA) under dataset name “MARROW payload”. Investigators can request access to the astronaut data at jsc-lsda@mail.nasa.gov.

## Ethics statement

The studies involving human participants were reviewed and approved by NASA Human Research Multilateral Review Board Johnson Space Center Institutional Review Board European Space Agency Medical Board Japanese Aerospace Exploration Agency Ottawa Health Science Network Research Ethics Board. The patients/participants provided their written informed consent to participate in this study.

## Author contributions

GT and OL participated in the concept and design. All authors participated in the acquisition, analysis, or interpretation of data. DS, GT, OL participated in the drafting of the article. All authors participated in the critical revision of the article for important intellectual content. DS and OL participated in the statistical analysis. GT and OL participated in the funding acquisition. All authors contributed to the article and approved the submitted version.

## Supplementary material

Supplementary Figure 1 Astronaut sample inventory. Twenty astronauts listened to an informed consent briefing session approximately one year before an astronaut’s scheduled flight. Fourteen astronauts, 11 men and 3 women consented to participating in the study. With 10 time-points () and 14 astronauts’, there were 140 potential blood samples for collection. One sample was not collected leaving 139 blood samples for RNA-sequencing. RNA quality control excluded 67 samples (RIN <8.0) and one sample was removed as an outlier (**Supplementary Figure 2**), leaving 71 samples for analysis *in silico*.

Supplementary Figure 2 Pre-flight sample outlier. Principal component analysis (PCA) of all 72 samples passing RNA quality metrics (RIN ≥8.0). Sample principal component scores were calculated from the variance stabilizing transformation (VST) of normalized read counts for the 59,901 genes. Each point represents an astronaut RNA sample () and colors indicate the time-point for sample collection ().

Supplementary Figure 3 Gene cluster dendrogram of the 276 differentially expressed genes identified from the temporal analysis leukocyte transcriptomes. The Euclidean distance was calculated between each of the 276 gene candidates using their z-scores scaled normalized read counts, which were then hierarchically clustered into the resulting tree dendrogram revealing two distinct gene clusters characterized by similar patterns of expression changes throughout the study. The horizontal red line represents where the static tree cut was made to separate and define the two clusters of differentially expressed genes across time. Each cluster is represented in the colored bar and identified by cluster number ().

Supplementary Figure 4 Expression profile of 15,410 genes expressed before, during and after long-duration spaceflight. Relative gene expression levels for the profile of 15,410 expressed transcripts (genes with mean normalized read count >45) displayed as violin plots of scaled z-scores across time. Z-scores represent the average normalized read counts for the 14 astronauts scaled across the 15,410 genes at each individual time-point. Medians indicated by white squares and upper and lower quartiles indicated by black squares. Colors denote the study phase.
