# Supplementary figures and images for: The transcriptome response of astronaut leukocytes to long missions aboard the International Space Station reveals immune modulation
Source: Front Immunol. 2023 Jun 22;14:1171103. doi: 10.3389/fimmu.2023.1171103 (PMC10324659; doi:10.3389/fimmu.2023.1171103)

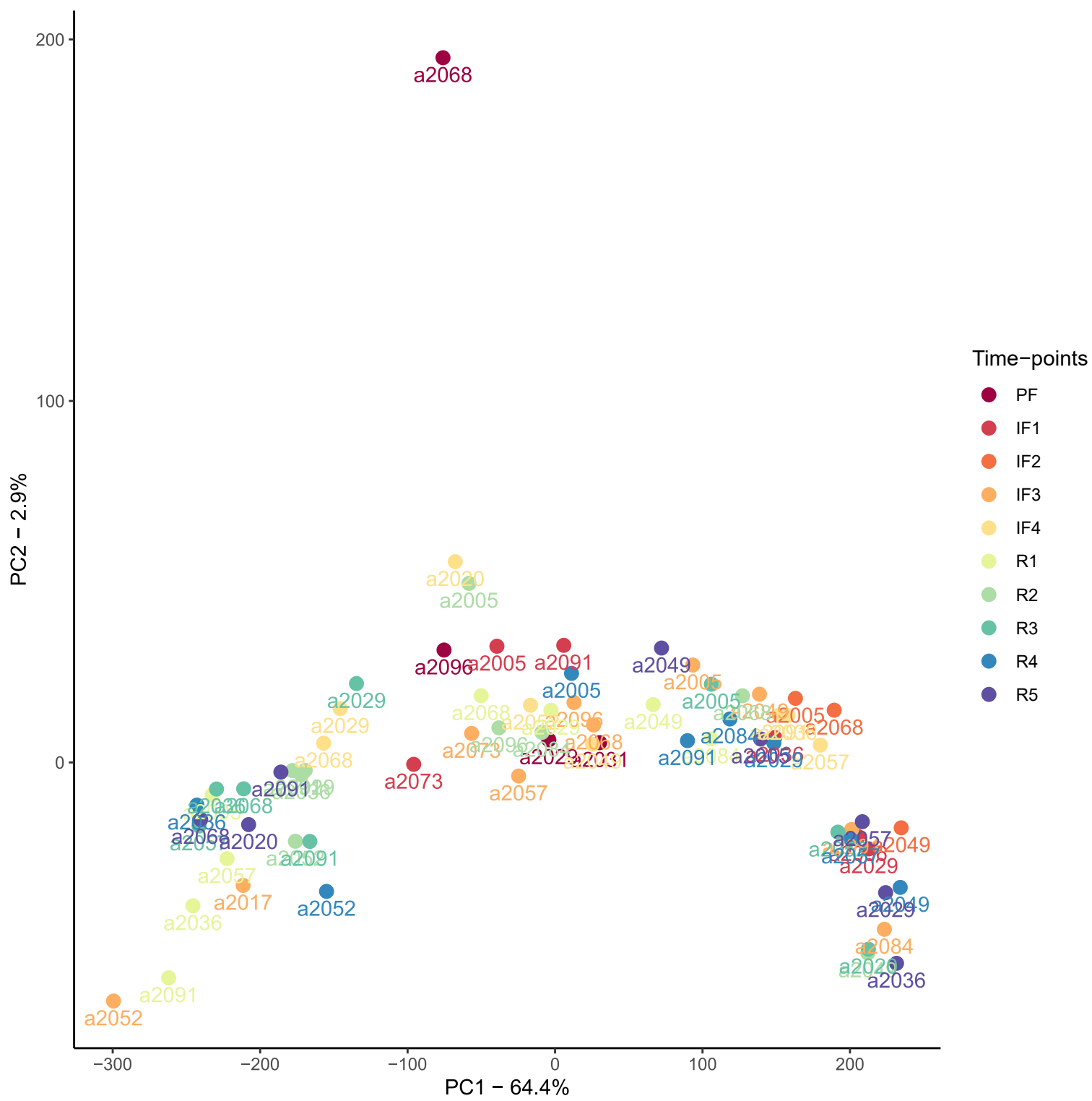

Supplement: Supplementary Figure 1 — Astronaut sample inventory. Twenty astronauts listened to an informed consent briefing session approximately one year before an astronaut’s scheduled flight. Fourteen astronauts, 11 men and 3 women consented to participating in the study. With 10 time-points () and 14 astronauts’, there were 140 potential blood samples for collection. One sample was not collected leaving 139 blood samples for RNA-sequencing. RNA quality control excluded 67 samples (RIN <8.0) and one sample was removed as an outlier ( Supplementary Figure 2 ), leaving 71 samples for analysis in silico. [file DataSheet_1.pdf]

# Cluster Dendrogram

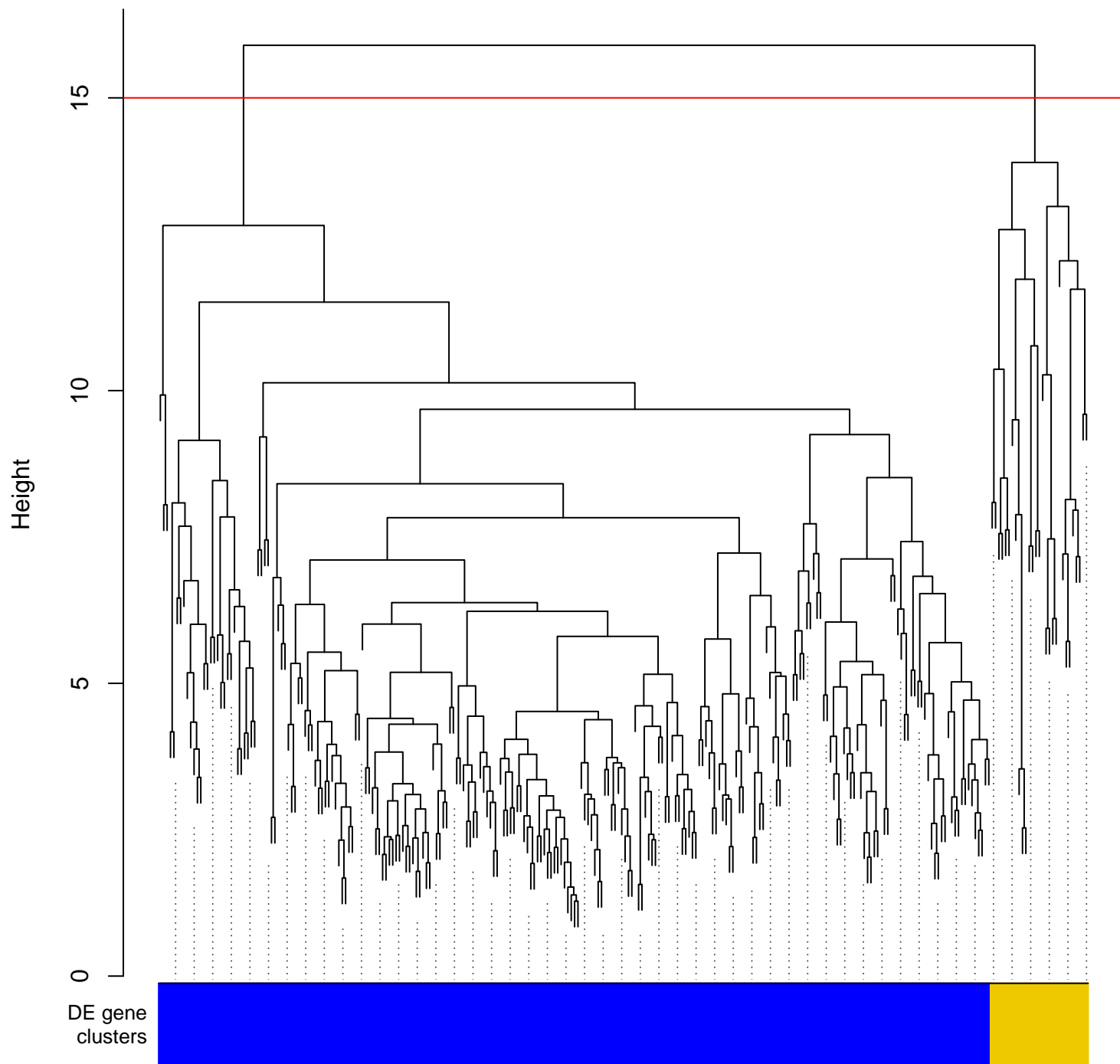

Supplement: Supplementary Figure 2 — Pre-flight sample outlier. Principal component analysis (PCA) of all 72 samples passing RNA quality metrics (RIN ≥8.0). Sample principal component scores were calculated from the variance stabilizing transformation (VST) of normalized read counts for the 59,901 genes. Each point represents an astronaut RNA sample () and colors indicate the time-point for sample collection (). [file DataSheet_2.pdf]

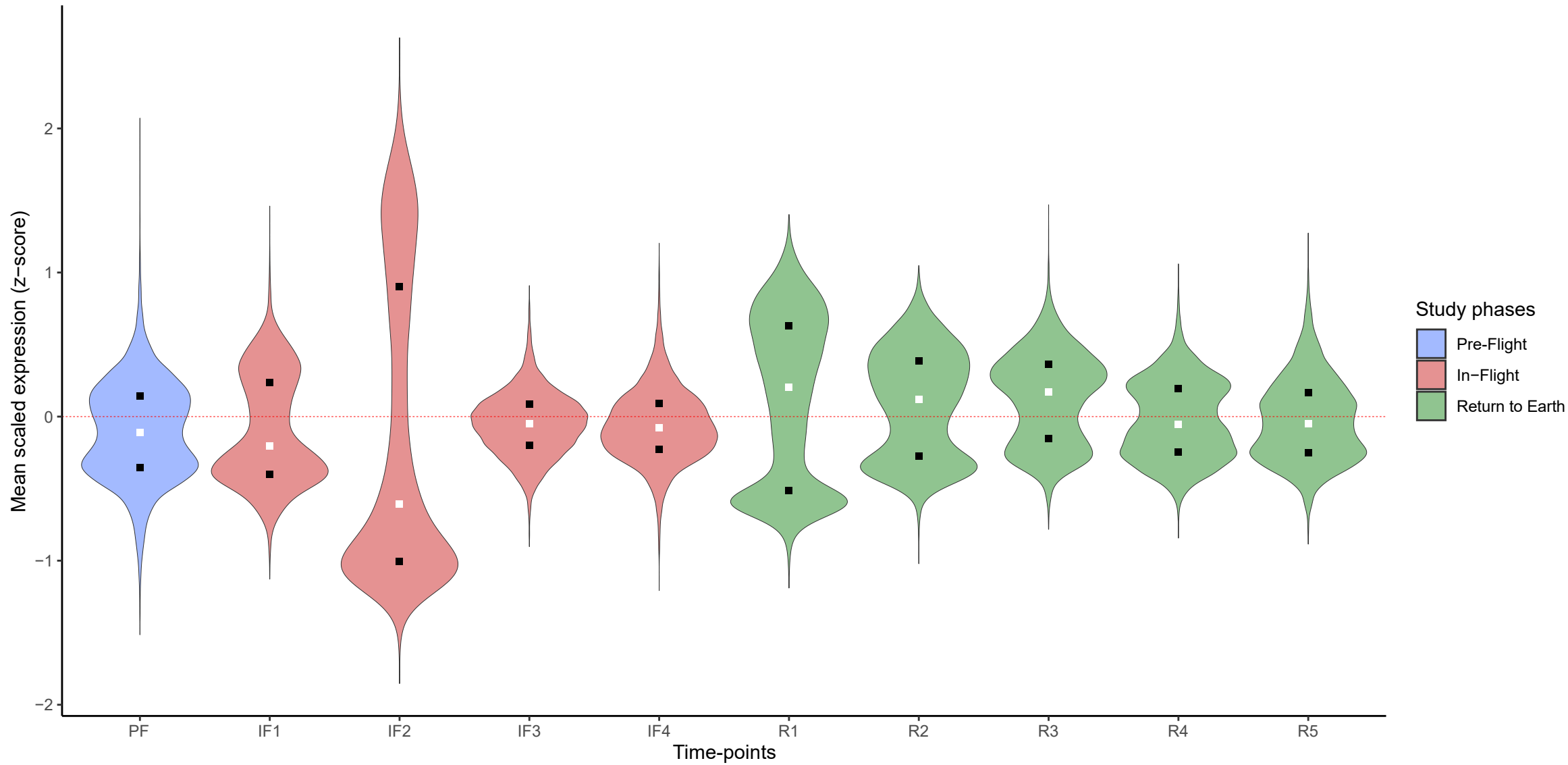

Supplement: Supplementary Figure 3 — Gene cluster dendrogram of the 276 differentially expressed genes identified from the temporal analysis leukocyte transcriptomes. The Euclidean distance was calculated between each of the 276 gene candidates using their z-scores scaled normalized read counts, which were then hierarchically clustered into the resulting tree dendrogram revealing two distinct gene clusters characterized by similar patterns of expression changes throughout the study. The horizontal red line represents where the static tree cut was made to separate and define the two clusters of differentially expressed genes across time. Each cluster is represented in the colored bar and identified by cluster number (). [file DataSheet_3.pdf]

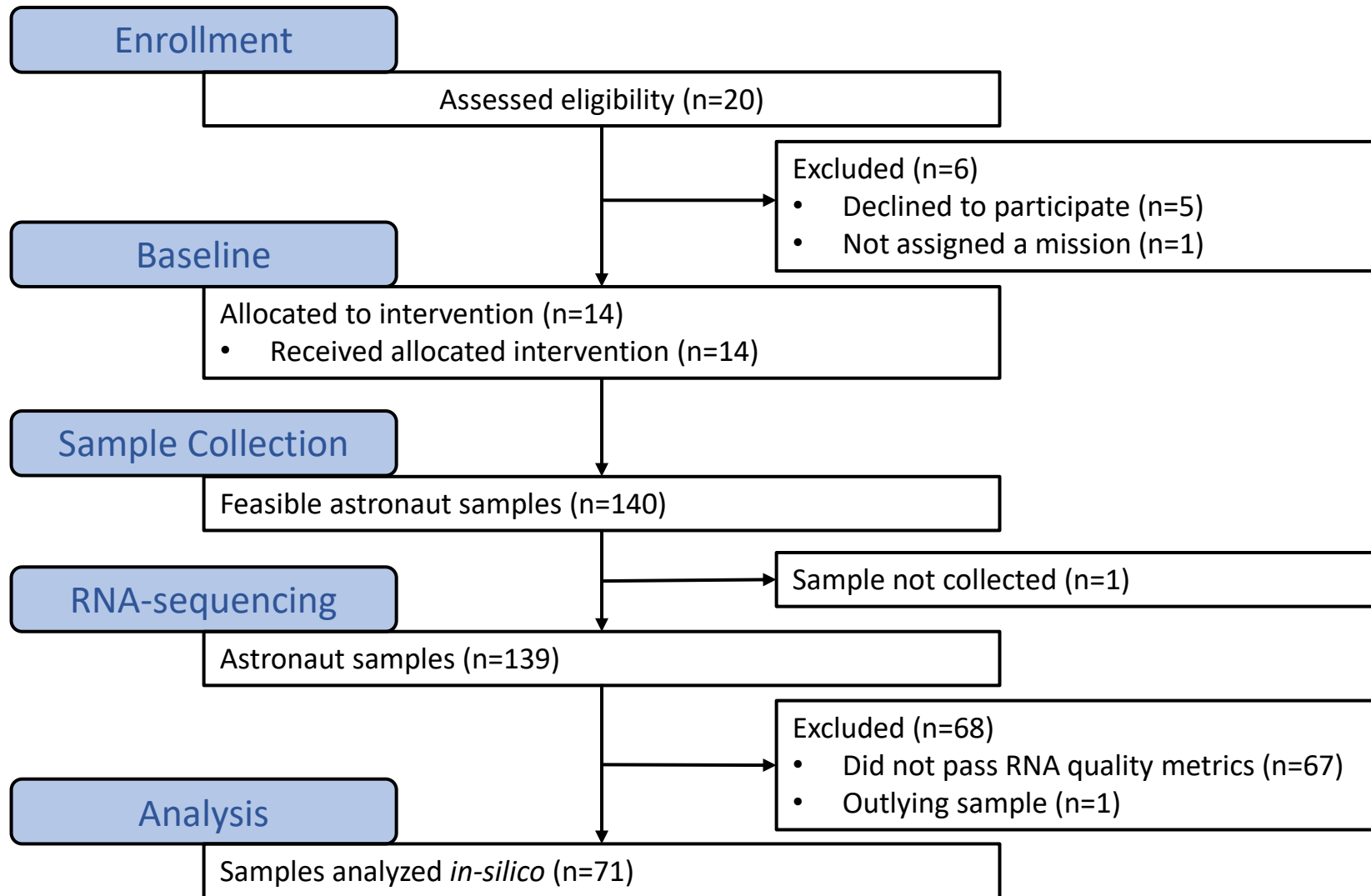

Supplement: Supplementary Figure 4 — Expression profile of 15,410 genes expressed before, during and after long-duration spaceflight. Relative gene expression levels for the profile of 15,410 expressed transcripts (genes with mean normalized read count >45) displayed as violin plots of scaled z-scores across time. Z-scores represent the average normalized read counts for the 14 astronauts scaled across the 15,410 genes at each individual time-point. Medians indicated by white squares and upper and lower quartiles indicated by black squares. Colors denote the study phase. [file DataSheet_4.pdf]
