# Supplementary material for: The transcriptome response of astronaut leukocytes to long missions aboard the International Space Station reveals immune modulation
Source: Front Immunol. 2023 Jun 22;14:1171103. doi: 10.3389/fimmu.2023.1171103 (PMC10324659; doi:10.3389/fimmu.2023.1171103)
Supplement: Supplementary file 5 [file Table_1.docx]

**Supplementary Table 1 |** Scaled expression summary statistics of 15,410 expressed genes.

| **Time-point** | **Mean scaled expression (z-score)** | | | | | | |
| --- | --- | --- | --- | --- | --- | --- | --- |
|  | **Min** | **Max** | **Max-Min** | **25% quartile** | **Median** | **75% quartile** | **IQR**^1^ |
| PF | -1.51 | 2.07 | 3.58 | -0.35 | -0.11 | 0.14 | 0.49 |
| IF1 | -1.13 | 1.46 | 2.59 | -0.40 | -0.20 | 0.24 | 0.64 |
| IF2 | -1.85 | 2.63 | 4.48 | -1.01 | -0.61 | 0.90 | 1.91 |
| IF3 | -0.90 | 0.91 | 1.81 | -0.20 | -0.05 | 0.09 | 0.29 |
| IF4 | -1.21 | 1.20 | 2.41 | -0.23 | -0.08 | 0.09 | 0.32 |
| R1 | -1.19 | 1.40 | 2.59 | -0.51 | 0.20 | 0.63 | 1.14 |
| R2 | -1.02 | 1.05 | 2.07 | -0.27 | 0.12 | 0.39 | 0.66 |
| R3 | -0.78 | 1.47 | 2.25 | -0.15 | 0.17 | 0.36 | 0.51 |
| R4 | -0.84 | 1.06 | 1.90 | -0.25 | -0.05 | 0.19 | 0.44 |
| R5 | -0.86 | 1.27 | 2.13 | -0.25 | -0.05 | 0.17 | 0.42 |

^1^Inter-quartile range.
